# The prevalence of psychiatric symptoms of pregnant and non-pregnant women during the COVID-19 epidemic

**DOI:** 10.1038/s41398-020-01006-x

**Published:** 2020-09-19

**Authors:** Yongjie Zhou, Hui Shi, Zhengkui Liu, Songxu Peng, Ruoxi Wang, Ling Qi, Zezhi Li, Jiezhi Yang, Yali Ren, Xiuli Song, Lingyun Zeng, Wei Qian, Xiangyang Zhang

**Affiliations:** 1 Shenzhen Kangning Hospital, Shenzhen, China; 2 Department of Clinial Psychology, Beijing Chao-Yang Hospital, Capital Medical University, Beijing, China; 3 CAS Key Laboratory of Mental Health, Institute of Psychology, Chinese Academy of Sciences, Beijing, China; 4 Department of Maternal and Child Health, Xiangya School of Public Health, Central South University, Changsha, China; 5 School of Medicine and Health Management, Tongji Medical College, Huazhong University of Science and Technology, Wuhan, China; 6 School of Health Science and Nursing, Wuhan Polytechnic University, Wuhan, China; 7 Department of Neurology, Ren Ji Hospital, Shanghai Jiao Tong University, School of Medicine, Shanghai, China; 8 Shenzhen Health Development Research Center, Shenzhen, China; 9 Liyuan Hospital of Tongji Medical College, Huazhong University of Science and Technology, Wuhan, China; 10 Department of Clinical Psychology, Yantai Affiliated Hospital of Binzhou Medical University, Yantai, China

**Keywords:** Depression, Human behaviour

## Abstract

Coronavirus disease 2019 (COVID-19) is rapidly spreading worldwide, with a staggering number of cases and deaths. However, available data on the psychological impacts of COVID-19 on pregnant women are limited. The purposes of this study were to assess the prevalence of psychiatric symptoms among pregnant women, and to compare them with non-pregnant women. From February 28 to March 12, 2020, a cross-sectional study of pregnant and non-pregnant women was performed in China. The online questionnaire was used to collect information of participants. The mental health status was assessed by patient health questionnaire, generalized anxiety disorder scale, insomnia severity index, somatization subscale of the symptom checklist 90, and post-traumatic stress disorder (PTSD) checklist-5. Totally, 859 respondents were enrolled, including 544 pregnant women and 315 non-pregnant women. In this study, 5.3%, 6.8%, 2.4%, 2.6%, and 0.9% of pregnant women were identified to have symptoms of depression, anxiety, physical discomfort, insomnia, and PTSD, respectively. However, the corresponding prevalence rates among non-pregnant women were 17.5%, 17.5%, 2.5%, 5.4%, 5.7%, respectively. After adjusting for other covariates, we observed that pregnancy was associated a reduced risk of symptoms of depression (OR = 0.23; 95% CI: 0.12–0.45), anxiety (OR = 0.26; 95% CI: 0.16–0.42), insomnia (OR = 0.19; 95% CI: 0.06–0.58), and PTSD (OR = 0.15; 95% CI: 0.04–0.53) during the COVID-19 epidemic. Our results indicate that during the COVID-19 epidemic in China, pregnant women have an advantage of facing mental problems caused by COVID-19, showing fewer depression, anxiety, insomnia, and PTSD symptoms than non-pregnant women.

## Introduction

In December 2019, unexplained pneumonia occurred in Wuhan, China and then became an epidemic. Now it is well-known as the 2019 coronavirus disease (COVID-19) epidemic, which is spreading rapidly around the world, causing a high incidence and heavy economic burden^1,2^. On January 7, 2020, the novel coronavirus was identified as the pathogen of the epidemic by genome sequencing^3^. Due to the rapid spread of the epidemic in many countries, the Emergency Committee of the World Health Organization (WHO) called an emergency meeting on 30 January, declaring the novel coronavirus 2019 (SARS-CoV-2) epidemic as a Public Health Emergency of International Concern (PHEIC)^4^. Subsequently, the WHO officially named COVID-19 on February 11^5^. Moreover, accumulating evidence has shown that person-to-person transmission has occurred between close contacts^6,7^. According to the official website of the Chinese National Health Commission, as of February 28, 2020, China had 79,251 confirmed cases and 2835 deaths, with a fatality rate of 3.6%^8^.

As a major public health emergency, the COVID-19 outbreak not only poses a great threat to human life^9^, but also has a significant psychological impact on public health^10^. Wang et al. reported that 53.8% of the general public rated the psychological impact as moderate to severe^11^. Yang et al. conducted a survey of 396 teenagers aged 8–18 years during epidemic period, and found that 22.0% of them had anxiety disorders and 10.4% had severe depressive symptoms^12^. Lai et al. investigated 1257 heath care workers from fever clinics or hospital wards in China during the COVID-19 outbreak, and found that a considerable number of them had symptoms of depression, anxiety, insomnia, and distress^13^. However, to our best knowledge, no research has focused on the psychological impact of COVID-19 epidemic on pregnancy women. Previous studies have shown that pregnancy women who bear significantly psychological burden are more likely to develop mental disorders and impaired social function in the future^14,15^. Further, the mental problems during pregnancy can also adversely affect the cognitive, emotional, and physical development of children^16–18^. Therefore, more attentions should be paid to the mental health of this special population. Moreover, pregnant women are at high risk of SARS-CoV-2 infection due to suppression of immune system functions^19^. During the COVID-19 epidemic, it is difficult for pregnant women to receive routine prenatal examinations or delivery, which may further increase the psychological burden of pregnant women.

Recently, a retrospective review reported nine pregnant women contracted COVID-19 pneumonia in late pregnancy, and showed that almost half of patients (4/9) had preterm birth^20^, similar to previous studies that pregnant women infected with severe acute respiratory syndrome coronavirus (SARS-CoV) or middle east respiratory syndrome coronavirus (MERS-CoV) had an increased risk of adverse pregnancy outcomes, including preterm birth and neonatal death^21,22^. Therefore, during COVID-19 epidemic, pregnant women may face greater psychological pressure and more complicated psychological problems.

We conducted this mental health survey among pregnant and non-pregnant women in China during the COVID-19 outbreak to assess the prevalence of depression, anxiety, physical discomfort, insomnia, post-traumatic stress disorder (PTSD) in both groups, and to explore whether pregnant women have more serious mental symptoms than non-pregnant women.

## Materials and methods

### Study design and participants

A cross-sectional survey was performed to assess the psychological status of pregnant and non-pregnant women in several Maternal and Child Health Hospitals in Beijing during the epidemic of COVID-19. As the Chinese government requires the general public to stay at home, our study adopted anonymous online surveys to collect data. The link to the survey was disseminated through social networks and through notifications from the maternal and child health hospitals. The notice required participants to be pregnant or non-pregnant women of childbearing age. Pregnant and non-pregnant women were invited electronically through the widely used social media app Wechat (WeChat, Tencent Inc, China). Because the notifications were spread through the maternal and child health hospitals, and pregnant women paid close attention to the health of their fetuses, they had received antenatal check-ups on time. Therefore, during the COVID-19 epidemic, they may pay more attention to hospital information than non-pregnant women, resulting in more pregnant women participating in the study.

Data collection was completed during February 28–March 12, 2020. The present study was approved by the Institutional Review Board of the Institute of Psychology, Chinese Academy of Sciences. All participants provided the informed consent before participating in the study.

### Data collection

The structured questionnaire was used to collect socio-demographic data, mental health information, and additional information about COVID-19. Socio-demographic data included age, nationality, marital status, occupation, education, height, weight, place of residence, smoking and drinking, annual family income and history of chronic diseases. In addition, respondents were asked to answer whether they followed the epidemic information every day, and whether any of their relatives or friends were infected with COVID-19. The mental health status of the participants was assessed by the Chinese version of various scales, including the patient health questionnaire (PHQ-9), generalized anxiety disorder scale (GAD-7), insomnia severity index (ISI), somatization subscale of the symptom checklist 90 (SCL-90), and post-traumatic stress disorder checklist-5 (PCL-5).

### Depression

Our study used PHQ-9 to measure depressive symptoms. Each PHQ-9 question asks the respondents how many days they have experienced depressive symptoms in the past two weeks. The scores for each item are as follows: 0 point (not at all), 1 point (a few days), 2 points (more than half the days), and 3 points (almost every day). The total score of PHQ-9 ranges from 0 to 27 and is used to identify women who meet the criteria for depressive symptoms. The validity and reliability of the Chinese version of PHQ-9 were reported, with a sensitivity of 0.89 and a specificity of 0.97^23^. The Cronbach’s alpha value of this study was 0.87. In this study, women with a PHQ-9 score of more than 10 were considered to have depressive symptoms.

### Anxiety

The GAD-7 was used to assess the anxiety level of participants. GAD-7 has 7 items, and each item is scored on a 4-point scale ranging from 0 to 3. The total score ranges from 0 to 21, and the higher score reflects the increased risk of GAD. GAD-7 has been previously validated among pregnant Chinese women^24^. Participants have anxiety symptoms with a cutoff of 7 or higher^24^. In this study, the Cronbach’ s alpha value was 0.93.

### Somatic symptoms

The SCL-90 somatization subscale was applied to measure the severity of somatic symptoms. The reliability and validity of the Chinese version of the SCL-90 have been established in previous studies^25^. It has 12 items, mainly reflecting the subjective physical discomfort over the past week. Each question is rated on a 5-point Likert scale. The total score of the subscale is between 12 and 60. An individual with a cutoff of 36 or higher indicates that he or she has obvious somatic symptoms. In this study, the Cronbach’s alpha coefficient of the scale was 0.94.

### Insomnia

Insomnia was assessed by the Chinese version of the 7-item ISI. Participants were asked to answer these questions on a 5-point scale, with a score range of 0–4. The total score ranges from 0 to 28. A cutoff of 15 or higher indicates insomnia symptoms. The tool has been proven to have good reliability and validity^26^. In this study, the Cronbach’s alpha coefficient of this scale was 0.93.

### Post-traumatic stress disorder (PTSD)

PTSD was evaluated by using the PTSD Checklist-5 (PCL-5). PCL-5 includes 20 items that reflect the standards of Diagnostic and Statistical Manual of Mental Disorders (DSM-IV). Participants were asked how troubled they were with each item in the past month, on a 0–4 Likert scale. The total score ranges from 0 to 80, and a score of 33 or higher indicates the presence of PTSD. In this study, the Cronbach’s alpha value was 0.96.

### Statistical analysis

Continuous variables were expressed as mean ± standard deviation (SD) and compared using Student’s *t*-tests. Due to non-normal distributions, the scores of the 5 measurement scales were expressed as the median of interquartile ranges (IQRs), and the difference in symptom scores between the two groups was compared by Mann–Whitney *U* test. Categorical data was expressed as a percentage and compared by chi-square test. Multivariable logistical regression was used to measure the independent association between pregnancy and mental health status. Statistical significance was evaluated at 5% level (two-tailed test). All analyses were performed using SPSS software version 18.0 (SPSS, Chicago, IL, USA).

## Results

### Characteristics of study population

Totally, we received 873 completed questionnaires, and 14 respondents with a diagnosis of mental illness were excluded from this study. Finally, 859 women (544 pregnant and 315 non-pregnant women) were included in the analysis. They were from 41 cities in China. Since the link to the survey was spread through social networks and through notifications from several maternal and child health hospitals in Beijing, three quarters of them came from Beijing. However, due to the traditional Chinese festival “Spring Festival” at the end of January, most of outsiders had left Beijing to be reunited with their families. Some people also participated in this study through the Internet link disseminated by the hospitals, resulting in our subjects coming from 40 cities in China except for Beijing. In addition, the participants had an age range of 20–47 years, with Han nationality accounting for 92.1%, and the married rate of 93.2%.

Table 1 compares the socio-demographic and behavior characteristics of pregnant and non-pregnant women. Pregnant women were more likely to be younger, get married, have higher weight and body mass index (BMI) and have a job than non-pregnant women. On the contrary, non-pregnant women had a higher proportion of current smokers, drinkers, and low family incomes than pregnant women. In addition, the proportion of pregnant women suffering from chronic diseases was significantly higher than that of non-pregnant women. However, there was no significant difference between pregnant and non-pregnant women in terms of nationality, education, height, and attention to epidemic information.

**Table 1:** Comparison of demographic and behavior characteristics of participators.

Characteristics	Control (*n* = 315)	Pregnancy women (*n* = 544)	*t/* *x*^2^	*p*-value
Age (years)	35.4 ± 5.7	31.1 ± 3.9	12.04	<0.001
Han nationality	292 (92.7)	499 (91.7)	0.26	0.612
Married	263 (83.5)	538 (98.9)	75.19	<0.001
Educational level (years)			0.93	0.052
High school and below	51 (16.2)	57 (10.5)		
College	214 (67.9)	396 (72.8)		
Bachelor or above	50 (15.9)	91 (16.7)		
Height (cm)	163.0 ± 5.0	162.7 ± 5.1	0.98	0.328
Weight (kg)	58.8 ± 8.3	67.0 ± 10.8	12.33	<0.001
BMI	22.1 ± 2.9	25.3 ± 4.0	13.46	<0.001
Employed	214 (67.9)	408 (75.0)	4.98	0.026
Smoking status (%)			38.39	<0.001
Nonsmoker	301 (95.6)	510 (93.8)		
Ex-smoker	3 (1.0)	34 (6.3)		
Current smokers	11 (3.5)	0 (0.0)		
Drinking status (%)			81.26	<0.001
Nonalcohol	249 (79.0)	485 (89.2)		
Abstainer	10 (3.2)	50 (9.2)		
Drinker	56 (17.8)	9 (1.7)		
Income			9.30	0.010
<8	89 (28.3)	105 (19.3)		
8–30	171 (54.3)	326 (59.9)		
>30	55 (17.5)	113 (20.8)		
Chronic diseases	50 (15.9)	120 (22.1)	4.81	0.028
Focusing on epidemic	278 (88.3)	456 (83.8)	3.15	0.076

*BMI* body mass index.

### Scores of measurements of psychiatric symptoms among pregnant and non-pregnant women

The median (IQR) scores of PHQ-9, GAD-7, SCL-90 somatization subscale, ISI, and PCL-5 in pregnant women were 2.0 (0.0–7.0), 0.0 (0.0–5.0), 15.0 (13.0–17.0), 2.0 (0.0–6.0), and 1.0 (0.0–5.0), respectively (Table 2). The corresponding median (IQR) scores of non-pregnant women were 3.0 (0.0–7.0), 2.0 (0.0–5.0), 15.0 (13.0–18.0), 3.0 (0.0–7.0) and 5.0 (1.0–14.0), respectively. Compared with non-pregnant women, pregnant women had lower scores of symptoms of depression, anxiety, and PTSD (all *p* < 0.05). However, there was no significant difference in SCL-90 somatic symptoms and insomnia symptoms between the two groups.

**Table 2:** Scores of measurements of psychiatric symptoms among participators.

Scales	Control (*n* = 315)	Pregnancy women (*n* = 544)	*p*-value
PHQ-9, depression symptoms	3.0 (0.0–7.0)	2.0 (0.0–7.0)	<0.001
GAD-7, anxiety symptoms	2.0 (0.0–5.0)	0.0 (0.0–5.0)	<0.001
Somatization subscale of SCL-90, somatic symptoms	15.0 (13.0–18.0)	15.0 (13.0–17.0)	0.364
ISI, insomnia symptoms	3.0 (0.0–7.0)	2.0 (0.0–6.0)	0.358
PCL-5, PTSD symptoms	5.0 (1.0–14.0)	1.0 (0.0–5.0)	<0.001

*PHQ-9* patient health questionnaire, *GAD-7* generalized anxiety disorder, *SCL-90* symptom checklist 90, *ISI* insomnia severity index, *PCL-5* post-traumatic stress disorder checklist-5.

In addition, we analyzed the relationships between social-demographic factors (education, income, job occupation) and the scores of psychiatric conditions in the pregnant group, non-pregnant group, and the whole participants. However, there was no significant difference between these social-demographic factors and the scores of psychiatric conditions except for the scores of somatization symptoms among the participants with different education levels and occupations in the whole participants.

### Comparison of prevalence of psychiatric symptoms among pregnant and non-pregnant women

Among the 315 non-pregnant women, 17.5% (55/315) had depression symptoms and 17.5% (55/315) had anxiety symptoms, which were significantly higher than those of pregnant women (5.3% and 6.8%, respectively; both *p* < 0.05). Moreover, insomnia (5.4% vs. 2.6%, *p* < 0.05) and PTSD (5.7% vs. 0.9%, *p* < 0.05) were more common in non-pregnant women than those in pregnant women. However, there was no significant difference in somatic symptoms between the two groups (2.5% vs. 2.4%, *p* > 0.05). After adjusting other covariables with statistical significance in univariate analysis, including age, married status, weight, BMI, employment, smoking, drinking, annul family income, and history of chronic diseases, we observed that pregnancy was associated with a reduced risk of depression (OR = 0.23; 95% CI: 0.12–0.45; *p* < 0.001), anxiety (OR = 0.26; 95% CI: 0.16–0.42; *p* < 0.001), insomnia (OR = 0.19; 95% CI: 0.06–0.58; *p* = 0.003) and PTSD (OR = 0.15; 95% CI: 0.04–0.53; *p* = 0.003) (Table 3). However, after adjusting for other variables, there was no significant difference in the prevalence of somatic symptoms between the two groups.

**Table 3:** Comparison of psychiatric symptoms among pregnant and non-pregnant women.

Outcomes	Control (*n* = 315)	Pregnancy women (*n* = 544)	OR (95% CI)	*p*-value^#^
Depression^*^	55 (17.5)	29 (5.3)	0.23 (0.12–0.45)	<0.001
Anxiety disorder^*^	55 (17.5)	37 (6.8)	0.26 (0.16–0.42)	<0.001
Somatic symptoms	8 (2.5)	13 (2.4)	0.57 (0.17–1.89)	0.359
Insomnia^*^	17 (5.4)	14 (2.6)	0.19 (0.06–0.58)	0.003
PTSD^*^	18 (5.7)	5 (0.9)	0.15 (0.04–0.53)	0.003

*PTSD* post-traumatic stress disorder.

^*^P < 0.05 in chi-square tests. ^#^Adjusting age, marital status, weight, BMI, employment status, smoking status, drinking status, family annul income, chronic diseases.

## Discussion

To the best of our knowledge, this study is the first investigation to assess and compare the prevalence of psychiatric symptoms including depression, anxiety, somatic symptoms, insomnia, and PTSD between pregnant and non-pregnant women during COVID-19 epidemic. Interestingly, our study showed that pregnant women had fewer psychiatric symptoms including depression, anxiety, insomnia, and PTSD than non-pregnant women.

In the current study, a small percentage of pregnant women displayed symptoms of depression, anxiety, physical discomfort, insomnia, and PTSD during the COVID-19 epidemic. However, a study by Hawryluck et al. showed that the detection rates of depressive symptoms and PTSD were 31.2% and 28.9%^27^, respectively, during the epidemic of severe acute respiratory syndrome (SARS) in 2003, which were significantly higher than our survey results in this study. Compared with COVID-19, more serious psychological effects may be attributed to higher mortality of SARS. Moreover, the Chinese government has taken many effective measures to control the further spread of the epidemic, such as city closure, traffic control, and self-isolation, which have enhanced public confidence in winning the fight against the epidemic. A study recently published by Lai et al. showed that 50.4%, 44.6%, 34.0%, and 71.5% of medical staffs reported symptoms of depression, anxiety, insomnia, and distress, respectively^13^. Another study by Wang et al. reported that 16.5% of the respondents had moderate to severe depressive symptoms, 28.8% had moderate to severe anxiety symptoms, and 8.1% had moderate to severe stress levels^11^. The reported prevalence rate was significantly higher than that in our current study. The differences in prevalence estimates may be explained by the variation of the study population and the different epidemic stages. Lai et al.’s study focused on medical health workers in fever clinics or hospital wards that received and treated COVID-19 infected patients, because their frequent contact with COVID-19 patients increased the risk of infection, leading to more serious mental problems^13^. On 23 January, 2020, Wuhan, as the origin of the epidemic in China, announced that it was closed, and the first-level public health emergency response was adopted in most provinces and cities. Wang et al. performed the survey on the initial stage of the epidemic, during 31 January to 2 February 2020, with daily surge of cases. The sudden outbreak of the epidemic made people feel fear and anxiety. However, our study was conducted from February 28 to March 12, 2020. This survey period corresponded to the reducing stage after the maximum point of the COVID-19 epidemic outbreak in China, and increasing people cured and disclosure of information on COVID-19 may alleviate the negative emotion among public.

Another finding of our study was that pregnant women had fewer psychiatric symptoms including depression, anxiety, insomnia, and PTSD than non-pregnant women. Similar results have been reported in previous studies. For example, Mota et al. reported that pregnant women were less likely to have depression than non-pregnant women^28^. Stampfel et al. found that pregnant women had a decreased risk of PTSD compared with non-pregnant women in the Hispanic and black populations^29^. However, Leach et al. reported that the levels of depression, anxiety, and stress of pregnant and non-pregnant women were similar^30^. In addition, contrary findings have been reported. For example, Adewuya et al. reported that anxiety disorders were more common among pregnant women (39.0% vs. 16.3%) than non-pregnant women^31^, which was confirmed by Uguz et al. ^32^. This discrepancy in results may be due to differences in the study population, diagnostic tool and criteria, and the impact of the COVID-19 epidemic. Compared with pregnant women, non-pregnant women may have fewer opportunities to keep in touch with medical staff, and are more disappointed with limited activities because of self-isolation.

As for the decreased risk of psychological effects on pregnant women, there may be several reasons. First, pregnant women decided to become pregnant when they had better mental health and financial situation. Therefore, they had better health mental condition than non-pregnant women. Second, pregnant women, as the focus of family attention, may obtain more emotional support from family members during COVID-19 epidemic. Third, increased contact with medical workers can provide support and decrease stress symptoms. Fourth, pregnant women reduce the processing of chemosensory anxiety signals, thereby reducing the perception of anxiety^33^. In addition, there is accumulating evidences that elevated hormone levels during pregnancy reduced the symptoms of PTSD^34^.

This study has several limitations. The first limitation is that our study is a cross-sectional survey and lacks longitudinal follow-up, which prevents us from exploring its impact on pregnancy outcomes. Therefore, the long-term psychological impact on this population is worth further investigation. Second, most participants were from Beijing, limiting the generalization of our findings to other regions. Third, our study did not collect data on psychological interventions for pregnant women, because hospitals can provide pregnant women with online health lecture on depression and anxiety, which may affect our results to a certain extent.

## Conclusion

Current study indicates that during the COVID-19 epidemic in China, pregnant women had a reduced risk of depression, anxiety, insomnia, and PTSD. However, these symptoms were more common among non-pregnant women of childbearing age, and the mental health of non-pregnant women was even more important than that of pregnant women. Our findings contribute to rationally allocate medical resources, and establish targeted psychological interventions for women of childbearing age to improve mental health during the COVID-19 epidemic.

## References

[1] Wu Z, McGoogan JM (2020). Characteristics of and important lessons from the coronavirus disease 2019 (COVID-19) outbreak in China: summary of a report of 72,314 cases from the Chinese Center for Disease Control and Prevention. JAMA.

[2] Onder G, Rezza G, Brusaferro S (2020). Case-fatality rate and characteristics of patients dying in relation to COVID-19 in Italy. JAMA.

[3] Zhu N (2020). A novel coronavirus from patients with pneumonia in China, 2019. N. Engl. J. Med..

[4] World Health Organization. Statement on the second meeting of the International Health Regulations (2005) Emergency Committee regarding the outbreak of novel coronavirus (2019-nCoV). https://www.who.int/news-room/detail/30-01-2020-statement-on-the-secondmeeting-of-the-international-health-regulations-(2005)-emergency-committee-regarding-the-outbreakof-novel-coronavirus-(2019-ncov). Accessed 2 Feb 2020.

[5] World Health Organization. WHO Director-General’s remarks at the media briefing on 2019-nCoV on 11 February 2020. https://www.who.int/dg/speeches/detail/who-director-general-s-remarks-at-the-media-briefing-on-2019-ncov-on-11-february-2020. Accessed 11 Feb 2020.

[6] Rothe C (2020). Transmission of 2019-nCoV infection from an asymptomatic contact in Germany. N. Engl. J. Med..

[7] Li Q (2020). Early transmission dynamics in Wuhan, China, of novel coronavirus-infected pneumonia. N. Engl. J. Med..

[8] The National Health Commission of China. Updates on the novel coronavirus outbreak up to February 28, 2020. http://www.nhc.gov.cn/xcs/yqtb/202002/4ef8b5221b4d4740bda3145ac37e68ed.shtml. Accessed 29 Feb 2020.

[9] Wang C, Horby PW, Hayden FG, Gao GF (2020). A novel coronavirus outbreak of global health concern. Lancet.

[10] Kang L (2020). The mental health of medical workers in Wuhan, China dealing with the 2019 novel coronavirus. Lancet Psychiatry.

[11] Wang, C. et al. Immediate psychological responses and associated factors during the initial stage of the 2019 coronavirus disease (COVID-19) epidemic among the general population in China. *Int. J. Environ. Res. Public Health* **17**, 1729 (2020). 10.3390/ijerph17051729 PMC7084952 32155789

[12] Wang Y, Yang YY, Li SW, Lei XW, Yang YF (2020). Investigation on the status and influencing factors for depression symptom of children and adolescents with home quarantine during the prevalence of novel coronavirus pneumonia. Chin. J. Child Health Care.

[13] Lai J (2020). Factors associated with mental health outcomes among health care workers exposed to coronavirus disease 2019. JAMA Netw. Open.

[14] Gutierrez-Lobos K, Scherer M, Anderer P, Katschnig H (2002). The influence of age on the female/male ratio of treated incidence rates in depression. BMC Psychiatry.

[15] Posmontier B (2008). Functional status outcomes in mothers with and without postpartum depression. J. Midwifery Women’s Health.

[16] Wu Y (2020). Association of prenatal maternal psychological distress with fetal brain growth, metabolism, and cortical maturation. JAMA Netw. Open.

[17] Closa-Monasterolo R (2017). The effect of postpartum depression and current mental health problems of the mother on child behaviour at eight years. Matern. Child Health J..

[18] Verbeek T (2012). Postpartum depression predicts offspring mental health problems in adolescence independently of parental lifetime psychopathology. J. Affect. Disord..

[19] Okuyama M, Mezawa H, Kawai T, Urashima M (2019). Elevated soluble PD-L1 in pregnant women’s serum suppresses the immune reaction. Front. Immunol..

[20] Chen H (2020). Clinical characteristics and intrauterine vertical transmission potential of COVID-19 infection in nine pregnant women: a retrospective review of medical records. Lancet.

[21] Wong SF (2004). Pregnancy and perinatal outcomes of women with severe acute respiratory syndrome. Am. J. Obstet. Gynecol..

[22] Alfaraj SH, Al-Tawfiq JA, Memish ZA (2019). Middle east respiratory syndrome coronavirus (MERS-CoV) infection during pregnancy: report of two cases & review of the literature. J. Microbiol Immunol. Infect..

[23] Zhang YL (2013). Validity and reliability of patient health questionnaire-9 and patient health questionnaire-2 to screen for depression among college students in China. Asia Pac. Psychiatry.

[24] Tong X, An D, McGonigal A, Park SP, Zhou D (2016). Validation of the generalized anxiety disorder-7 (GAD-7) among Chinese people with epilepsy. Epilepsy Res..

[25] Chen G (2005). Psychological symptoms and nonfatal unintentional injuries among Chinese adolescents: a prospective study. J. Adolesc. Health.

[26] Gagnon C, Belanger L, Ivers H, Morin CM (2013). Validation of the Insomnia Severity Index in primary care. J. Am. Board Fam. Med..

[27] Hawryluck L (2004). SARS control and psychological effects of quarantine, Toronto, Canada. Emerg. Infect. Dis..

[28] Mota N, Cox BJ, Enns MW, Calhoun L, Sareen J (2008). The relationship between mental disorders, quality of life, and pregnancy: findings from a nationally representative sample. J. Affect. Disord..

[29] Stampfel CC, Chapman DA, Alvarez AE (2010). Intimate partner violence and posttraumatic stress disorder among high-risk women: does pregnancy matter?. Violence Women.

[30] Leach LS, Christensen H, Mackinnon A (2014). Pregnancy and levels of depression and anxiety: a prospective cohort study of Australian women. Aust. N. Z. J. Psychiatry.

[31] Adewuya AO, Ola BA, Aloba OO, Mapayi BM (2006). Anxiety disorders among Nigerian women in late pregnancy: a controlled study. Arch. Women’s Ment. Health.

[32] Uguz F, Gezginc K, Kayhan F, Sari S, Buyukoz D (2010). Is pregnancy associated with mood and anxiety disorders? A cross-sectional study. Gen. Hosp. Psychiatry.

[33] Lubke KT, Busch A, Hoenen M, Schaal B, Pause BM (2017). Pregnancy reduces the perception of anxiety. Sci. Rep..

[34] Smith MV, Poschman K, Cavaleri MA, Howell HB, Yonkers KA (2006). Symptoms of posttraumatic stress disorder in a community sample of low-income pregnant women. Am. J. Psychiatry.

